# Transcriptomic analysis of subarachnoid cysts of *Taenia solium* reveals mechanisms for uncontrolled proliferation and adaptations to the microenvironment

**DOI:** 10.1038/s41598-024-61973-9

**Published:** 2024-05-23

**Authors:** Miguel A. Orrego, Michal W. Szczesniak, Carlos M. Vasquez, Manuela R. Verastegui, Javier A. Bustos, Hector H. Garcia, Theodore E. Nash, Cysticercosis Working Group in Peru, Hector H. Garcia, Robert H. Gilman, Armando E. Gonzalez, Manuela Verastegui, Mirko Zimic, Javier Bustos, Seth E. O’Neal, Silvia Rodriguez, Isidro Gonzalez, Herbert Saavedra, Sofia Sanchez, Manuel Martinez, Saul Santivañez, Holger Mayta, Yesenia Castillo, Monica Pajuelo, Gianfranco Arroyo, Nancy Chile, Luz Toribio, Miguel A. Orrego, Maria T. Lopez, Luis Gomez, Cesar M. Gavidia, Ana Vargas-Calla, Eloy Gonzales, Luz M. Moyano, Ricardo Gamboa, Claudio Muro, Percy Vichez, Sukwan Handali, John Noh, Theodore E. Nash, Jon Friedland

**Affiliations:** 1 Laboratory of Immunopathology in Neurocysticercosis, Facultad de Ciencias e Ingenierías, Universidad Peruana Cayetano Heredia, Lima, Peru; 2 Institute of Human Biology and Evolution, Adam Mickiewicz University in Poznan, Poznan, Poland; 3 Department of Neurosurgery, Instituto Nacional de Ciencias Neurológicas, Lima, Peru; 4 Infectious Diseases Research Laboratory, Facultad de Ciencias e Ingenierías, Universidad Peruana Cayetano Heredia, Lima, Peru; 5 Cysticercosis Unit, Instituto Nacional de Ciencias Neurológicas, Lima, Peru; 6 Center for Global Health, Universidad Peruana Cayetano Heredia, Lima, Peru; 7 Universidad Peruana Cayetano Heredia, Lima, Peru; 8 Instituto Nacional de Ciencias Neurologicas, Lima, Peru; 9 School of Veterinary Medicine, Universidad Nacional Mayor de San Marcos, Lima, Peru; 10 Cysticercosis Elimination Program, Tumbes, Peru; 11 Centers for Disease Control, Atlanta, GA USA; 12 NIAID, NIH, Bethesda, MD USA; 13 St George’s University of London, London, UK

**Keywords:** Cell biology, Molecular biology, Neuroscience, Stem cells, Neurology

## Abstract

Subarachnoid neurocysticercosis (SANCC) is caused by an abnormally transformed form of the metacestode or larval form of the tapeworm *Taenia solium*. In contrast to vesicular parenchymal and ventricular located cysts that contain a viable scolex and are anlage of the adult tapeworm, the subarachnoid cyst proliferates to form aberrant membranous cystic masses within the subarachnoid spaces that cause mass effects and acute and chronic arachnoiditis. How subarachnoid cyst proliferates and interacts with the human host is poorly understood, but parasite stem cells (germinative cells) likely participate. RNA-seq analysis of the subarachnoid cyst bladder wall compared to the bladder wall and scolex of the vesicular cyst revealed that the subarachnoid form exhibits activation of signaling pathways that promote proliferation and increased lipid metabolism. These adaptions allow growth in a nutrient-limited cerebral spinal fluid. In addition, we identified therapeutic drug targets that would inhibit growth of the parasite, potentially increase effectiveness of treatment, and shorten its duration.

## Introduction

Neurocysticercosis (NCC) is the infection of the central nervous system (CNS) with the larval stage of the pork tapeworm *Taenia solium* and is the most important cause of acquired epilepsy worldwide^1–3^. The parasite has a complex life cycle that includes two mammalian hosts; humans are the only definitive hosts that harbor the adult tapeworm form of the parasite in their small intestine, which produce ten thousands of eggs in each parasite segment, which are excreted in the feces. The pig, the natural intermediate host, becomes infected with the cystic stage after ingestion of feces contaminated with *T. solium* ova. The liberated oncospheres cross the intestine, enter the bloodstream, and lodge in any vascular tissue, but mostly mature in the muscles and less so in the brain^1^. Humans can also host the larval stage and develop NCC.

After reaching the CNS, cysts can develop in parenchymal or extra parenchymal regions of the brain^4^. Subarachnoid NCC (SANCC) is the most severe form of the extra parenchymal NCC^4–6^. This abnormal cystic parasite is primarily located in the basal subarachnoid spaces or Sylvian fissures where they proliferate and develop into a multiple vesicle form, which is produced by the continuous proliferation of the bladder wall^7^. Subarachnoid cysts lack the usual metacestode structure, and a viable scolex is absent, although a degenerated scolex is sometimes present, resulting in an abnormal parasite incapable of developing into tapeworm after ingestion^6,7^. The continuous growth of the bladder wall commonly causes mass effects. The host eventually mounts a strong inflammatory response leading to arachnoiditis, fibrosis, thickening of the leptomeninges, hydrocephalus, intracranial hypertension, and a significant increase in mortality (20%)^8–10^.

Treatment of parenchymal NCC involves two effective anthelmintics drugs: praziquantel, albendazole, or a combination of both, but the destruction of the parasites results in the release of antigens that exacerbate the neuroinflammatory processes^2,4,11^. For this reason, current NCC treatment schemes include anti-inflammatory drugs, mainly corticosteroids, to reduce the detrimental effect of inflammation^12^. Moreover, SANCC is more difficult to treat than the parenchymal NCC^4^. Long-term anti-parasitic treatment is frequently required to treat SANCC and in many cases it fails to totally kill the parasite^4^. Unlike treatment of vesicular cysts, which requires a relatively brief course of therapy that initiates injury followed by a killing host inflammatory assault, treatment of SANCC is prolonged most likely because all viable cells, which are capable of regrowth, need to be eliminated^4^. Directed host inflammatory responses are also important. Corticosteroids are also immunosuppressive agents, which decrease effective host-parasite killing. Their use probably requires concomitant treatment with cysticidal agents to ensure the most effective treatment.

The natural development of cysts involves close host-parasite interactions, which allow development of the parasite in an organized and controlled manner, regulating its homeostasis and cellular processes^13^. Subarachnoid cysts develop in a particular microenvironment surrounded by cerebrospinal fluid (CSF), in contrast to parenchymal cysts (also referred to as vesicular cysts) that are in close contact with the brain substance and blood vessels^6,7,14^. The CSF does not supply the same nutrients or growth factors that are present in blood, so subarachnoid cysts likely acquire adaptations including those to secure sufficient nutrients and concomitant changes in gene expression supporting proliferation.

Identification of informative biomolecules that play a role in critical biological processes, through sequencing and/or identification of DNA, RNA, and proteins, gives rise to an understanding of the molecular basis of the normal development of many organisms as well as the alterations that promote several pathologies^15–20^. This knowledge has also allowed the identification of new therapeutic targets and bioactive drugs^21^. Although the subarachnoid cyst has a tumor-like behavior, the biological basis of its proliferation is poorly studied; however, the active participation of germinative (parasite stem cells) and the mitogen-activated protein kinases (MAPK) pathway in the abnormal proliferation of the bladder wall have been reported^22,23^.

In the present study, we sequenced total RNA from the bladder wall of vesicular cysts, the bladder wall of proliferating subarachnoid cysts, and the scolex of vesicular cysts to identify the molecular mechanisms associated with the continuous proliferation of the bladder wall and survival of the subarachnoid cysts.

## Results

First, we compared the transcriptomic profile of the bladder wall in both forms of cysticerci (Supplementary Fig. 1A) using high-throughput RNA sequencing (RNAseq). Over 33 million paired-end reads per library were obtained and aligned to the *T. solium* reference genome; 95.93% of reads mapped to the genome. From a total of 12,467 annotated genes, we observed changes in the expression levels of 88 transcripts (23 up- and 65 down-regulated) in the bladder wall of subarachnoid cysts compared to the same structure in the vesicular form (Supplementary Fig. 1B,C). TsM_001026100 (Transposon Ty3-G Gag-Pol protein; Log2FC: 2.00, *p*-adj: 0.008), TsM_001048700 (Basement membrane-specific heparin sulfate proteoglycan core protein; Log2FC: 1.99, *p*-adj: 0.001), and TsM_001023300 (alpha-Tubulin; Log2FC: 1.86, *p*-adj: 0.019) were the most overexpressed genes. Additionally, TsM_000338300 (tRNA splicing endonuclease; Log2FC: − 2.56, *p*-adj: 0.0003), TsM_000244100 (IQ domain-containing protein K; Log2FC: − 2.20, *p*-adj: 0.004), and TsM_000801500 (Unnamed protein product; Log2FC: − 2.13, *p*-adj: 0.003) were most down-expressed (Supplementary data 1).

Next, we compared the transcriptome profile between the bladder wall of the subarachnoid form and the scolex of the vesicular cyst (Supplementary Fig. 2A). Total RNA from each sample was isolated and sequenced under the same conditions. 96.14% of reads mapped to the reference genome, and we observed changes in the expression levels of 2,084 genes (930 up- and 1154 down-expressed) in the bladder wall of subarachnoid cysts compared to the scolex of the vesicular form (Supplementary Fig. 2B,C). TsM_000414500 (Non-translated sequence; Log2FC: 2.71, *p*-adj: 1.22 × 10^–25^), TsM_000708200 (SH2 domain-containing protein 5; Log2FC: 2.63, *p*-adj: 1.02 × 10^–19^), and TsM_000595700 (Unnamed protein product; Log2FC: 2.61, *p*-adj: 1.5 × 10^–19^) were the most overexpressed genes. Additionally, TsM_001075800 (Tetraspanin; Log2FC: − 4.09, *p*-adj: 7.32 × 10–75), TsM_000095600 (Ectonucleotide pyrophosphastase/phosphodiesterase; Log2FC: − 5.25, *p*-adj: 6.10 × 10^–64^), and TsM_000534500 (Unnamed protein product; Log2FC: − 5.71, *p*-adj: 8.58 × 10^–55^) were most down-expressed (Supplementary data 2).

### Subarachnoid cysts have alterations in signaling pathways that promote their uncontrolled proliferation and abnormal development

The ability of the subarachnoid cysts to expand, regrow, extend into contiguous spaces and seed the spinal cord, as well as the abnormal parasite morphology, are indicative of proliferation^6,7^. After GO analysis, we observed enrichment in signaling pathways that promote cell proliferation in the bladder wall of subarachnoid cysts compared to the vesicular cysts, including upregulation of receptor tyrosine-protein kinase erbB-3 (ERBB3) signaling pathway (GO: 1905580), transforming growth factor beta (TGF-β) receptor signaling pathway (GO: 0007179), cellular response to insulin-like growth factor stimulus (GO: 1990314) associated with biological process, and MAP kinase activity (GO: 0008349), G protein-coupled peptide receptor activity (GO: 0008528) with molecular function (Fig. 3D) (Supplementary datas 3, 4).

Other relevant results include increased activity of the biosynthetic pathway of nucleoside triphosphate process (GO: 0009142), enhancement of the neuropeptide signaling pathway (GO: 0007218), and response to norepinephrine (GO: 0071873), all grouped in biological process; integrin binding (GO: 0005178), platelet-derived growth factor binding (GO: 0048407), neuropeptide receptor activity (GO: 0008188), and vasopressin receptor activity (GO: 0005000) classified in molecular function. Also, the downregulation for anatomical structure development (GO: 0048856), regulation of cell cycle process (GO: 0010564), and cellular response to DNA damage stimulus (GO: 0006974).

**Figure 1:**
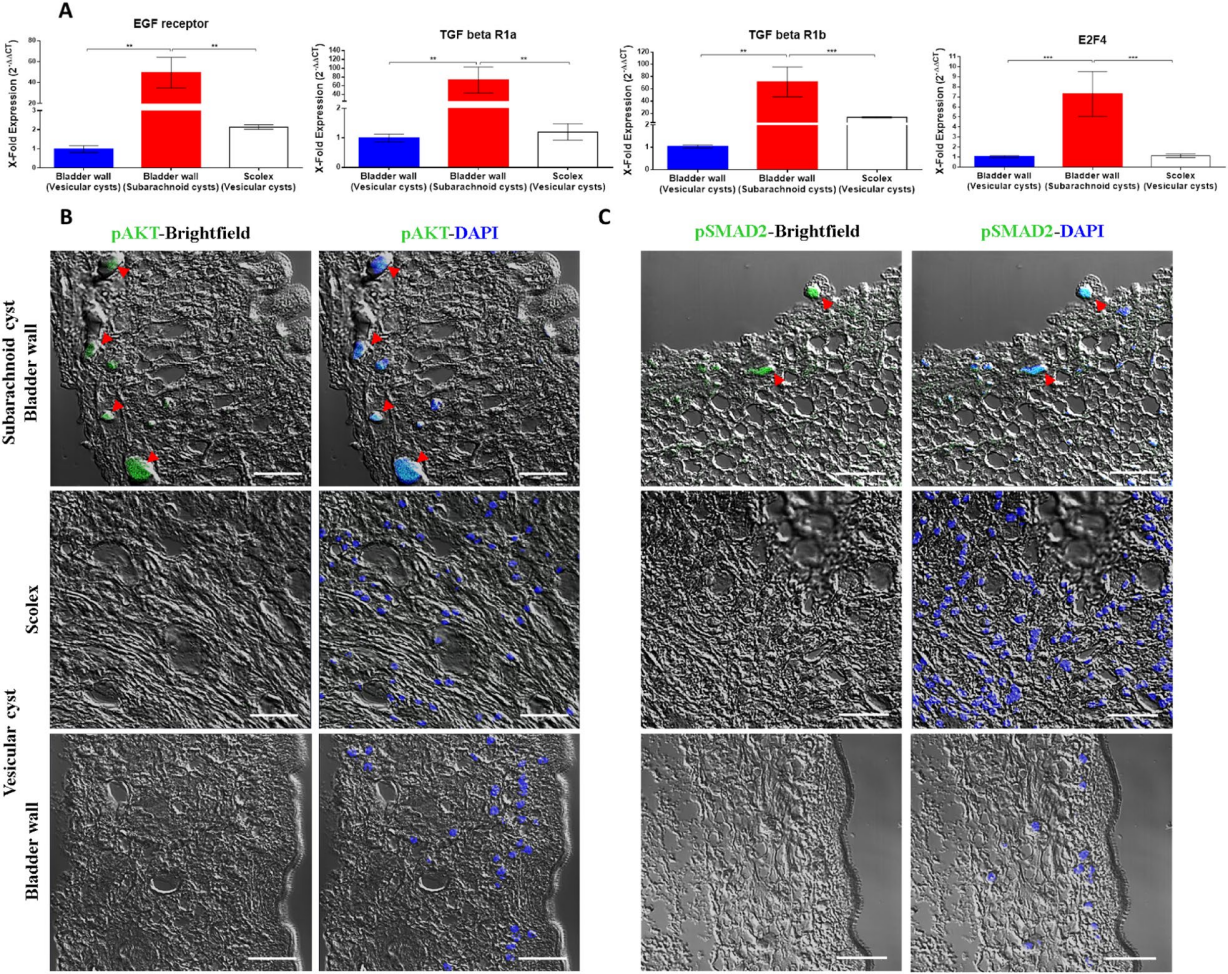
Over-activation of signaling pathways that promotes cell proliferation in the bladder wall of subarachnoid cysts. (**A**) Quantitative gene expression for *egf receptor*, *tgf-β receptor 1* isoforms, and transcription factor *e2f4* in subarachnoid and vesicular cysts by qPCR. Significant overexpression in the evaluated genes was observed in the subarachnoid cysts. Statistically significant differences are indicated by asterisks (Mann–Whitney U test). Asterisks represent level of significance: ***p < 0.001; **p < 0.01. (**B**) Localization of phospho-AKT and (**C**) Localization of phospho-SMAD2 in the bladder wall of the subarachnoid (upper) and vesicular cysts (middle and lower) by IF. Confocal microscopy images of representative samples; positive cells were observed only in the bladder wall of subarachnoid cysts (red arrowheads). White scale bar: 20 µm, ×20, zoom4.

ERBB3, a member of epidermal growth factor receptor (EGFR) family with tyrosine kinase activity, is associated with cell proliferation, differentiation, and migration^24^. After performing quantitative PCR, the expression of homolog *egfr* gene from *T. solium* (TsM_000399900, *p* value **: 0.0091) was significantly increased in the subarachnoid cysts compared with the vesicular cysts (Fig. 1A). After the ligand binds to the tyrosine kinase receptor, the principal intracellular signaling pathways include the mitogen-activated protein kinases (MAPK) and the phosphoinositide-3-kinase (PI3K)/AKT pathway. The role of the MAPK pathway in promoting the proliferation of the bladder wall of the subarachnoid cyst has been previously reported^23^, so we focused on evaluating the phosphorylation (activation) of the downstream protein AKT by IF, and we observed phospho-AKT positive cells only in the bladder wall of the subarachnoid cysts (Fig. 1B).

TGF-β, a member of a growth factors family expressed in vertebrates and invertebrates, shows a wide variety of effects on cells after interacting with its serine-threonine kinase receptor^25^. We validated the significant overexpression of the two isoforms of *tgf-β receptor 1* (TsM_000502400, *p* value **: 0.0022; TsM_000081300, *p* value **: 0.0091, ***:0.0004) and *transcription factor e2f4* (TsM_000874400, *p* value ***: 0.0004) by quantitative PCR (Fig. 1A). Next, we evaluated the phosphorylation (activation) of the intracellular mediator SMAD2 by IF and phospho-SMAD2 positive cells were observed only in the bladder wall of the subarachnoid cysts (Fig. 1C). These results suggest the proliferative nature of the bladder wall in subarachnoid cysts through the direct involvement of EGF and TGF-β signaling pathways.

The progression through stages and subsequent strobila development follows a well-organized pattern involving the establishment of an antero-posterior (AP) axis, driven by specific gene expression and pathways like WNT^26,27^. Studies in *Echinococcus multilocularis* and *Hymenolepis microstoma* demonstrate conserved expression patterns in several genes belonging to the WNT family during metacestode development^26^. For instance, *wnt1*, *wnt11a*, and *wnt11b* are expressed in the posterior domain, while *wnt2* is expressed in antero-lateral domains. Meanwhile, WNT5 is secreted in lateral domains to restrict the lateral expansion of tissues (mediolateral axis)^27^. Analysis of expression profile in subarachnoid cysts reveals alterations in homologous WNT ligands; we observed an overexpression of *wnt11b* (TsM_000542700; *p*-adj = 2.21 × 10^–7^) and down regulation of *wnt1* (TsM_000159300; *p*-adj = 0.0008, and 0.02) in the bladder wall of these cysts (Supplementary datas 1, 2). Additionally, there was a decrease in *wnt5* expression (TsM_000297700; *p*-adj = 0.006) (Supplementary data 1). These findings suggest that the abnormal growth of the posterior axis in subarachnoid cysts may be driven by WNT11B and WNT5 through a non-canonical pathway.

Although *wnt2* (TsM_000270800) and *wnt4* (TsM_000233500) also showed altered gene expression in the bladder wall of subarachnoid cysts compared to vesicular form, these changes were not statistically significant (Data not included).

Tumor necrosis factor-alpha (TNF-α) is one of the proinflammatory mediator expressed as an integral component of the host immune response during infection processes^28^. Prior studies in *Schistosoma mansoni* detected a gene encoding a TNF receptor and a description of its proposed downstream signaling pathway and biological effects upon activation^29^. We observed an overexpression of *tnf receptor* (TsM_000678000; *p*-adj = 0.03) in the bladder wall of subarachnoid cysts (Supplementary data 2).

## TGF-β and EGF stimulate the proliferation of germinative cells of subarachnoid cysts.

**Figure 2:**
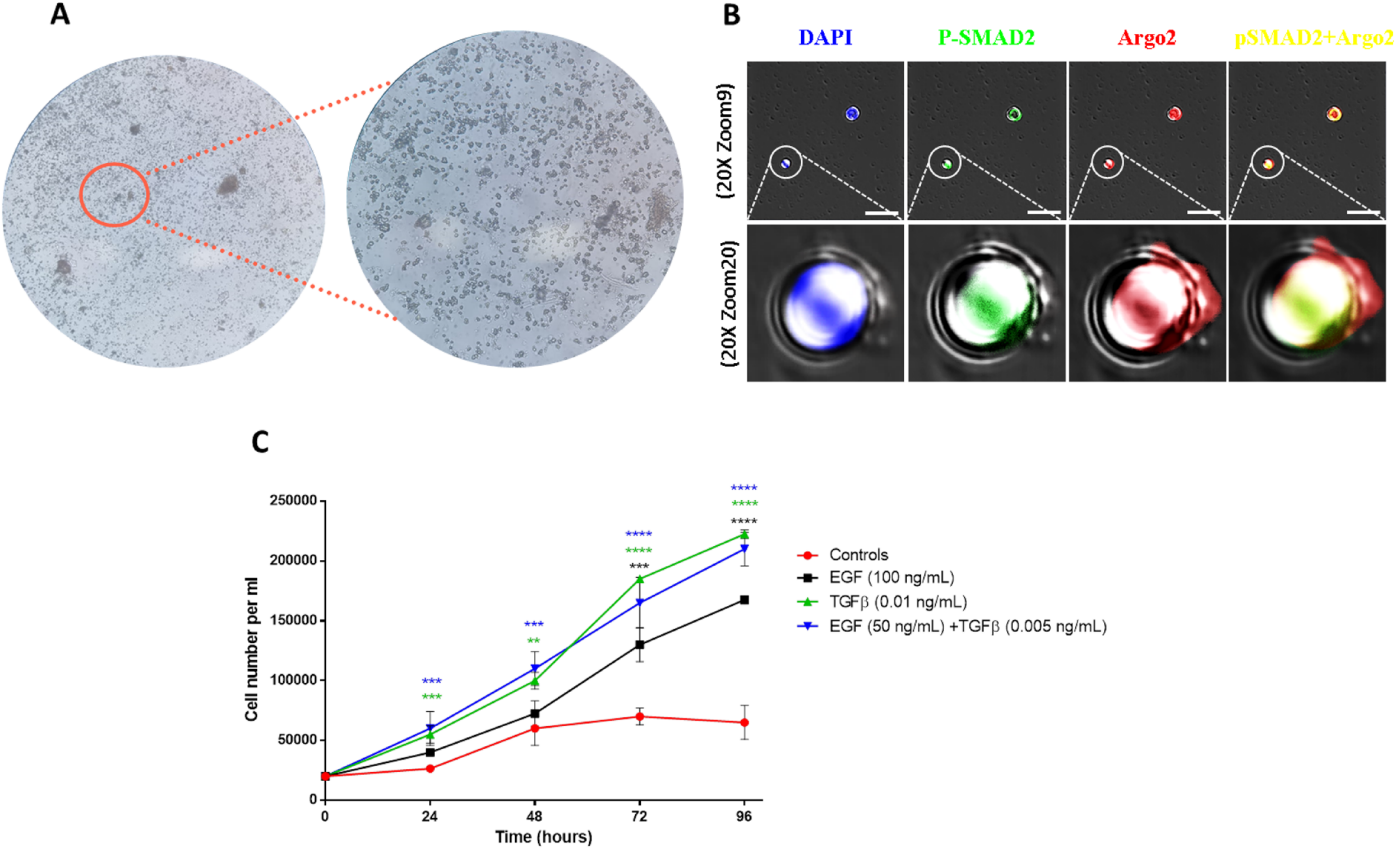
In vitro effects of EGF and TGF-β on germinative cells proliferation. (**A**) Representative images of germinative cells isolated from subarachnoid cysts and cultured for 15 days. (**B**) Characterization of cell cultures by FISH and IF. An LNA probe was designed to evaluate the expression of the germline marker *argo2* together with SMAD2 phosphorylation. Representative images of confocal microscopy; white scale bar: 10 µm. (**C**) Cell growth curves of germinative cells isolated from subarachnoid cysts. EGF at 100 ng/mL produced a significant increase in cells after 72 h (black asterisks), TGF beta at 0.01 ng/mL after 24 h (green asterisks). Finally, the combined use of EGF and TGF at half concentration produced a significant increase in cell growth after 24 h (blue asterisks). Asterisks indicate statistically significant differences between groups (treated and control). One-way ANOVA test (***p < 0.001; ****p < 0.0001).

Germinative cells, the undifferentiated stem cells of cestodes, play a pivotal role in the transition between the parasite’s life stages^30,31^. They are the only mitotically active and self-renewing cell population^31^. Previously, we successfully identified germinative cells within the subarachnoid cyst bladder^22^. Subsequently, we established theses germinative cells in culture and used them to explore and analyze host-parasite interactions^22,23^, including the direct effect of EGF and TGF-β. We confirmed the totipotency status of cell cultures by evaluating the expression of the Argo2 marker using FISH (Fluorescence In Situ Hybridization). Argo2 belongs to the Argonaute family of proteins and plays a crucial role in maintaining genomic integrity in the germline^32^. When TGF-β was added at a concentration of 0.01 ng/mL or EGF at 100 ng/mL, we observed a significant increase in cell count after 24 and 72 h respectively (*p* value ***: < 0.001 for TGF-β and EGF) (Fig. 2C). Notably, when we exposed the cells to half the concentration of each of the two growth factors, a continuous and significant increase in cell number was observed within 24 h (*p* value ***: < 0.001). These findings suggest that the continued proliferation of the bladder wall involves the active participation of germinative cells stimulated directly by host TGF-β and EGF.

### The bladder wall of the subarachnoid cysts preserves histological characteristics but with an abnormal organization

**Figure 3:**
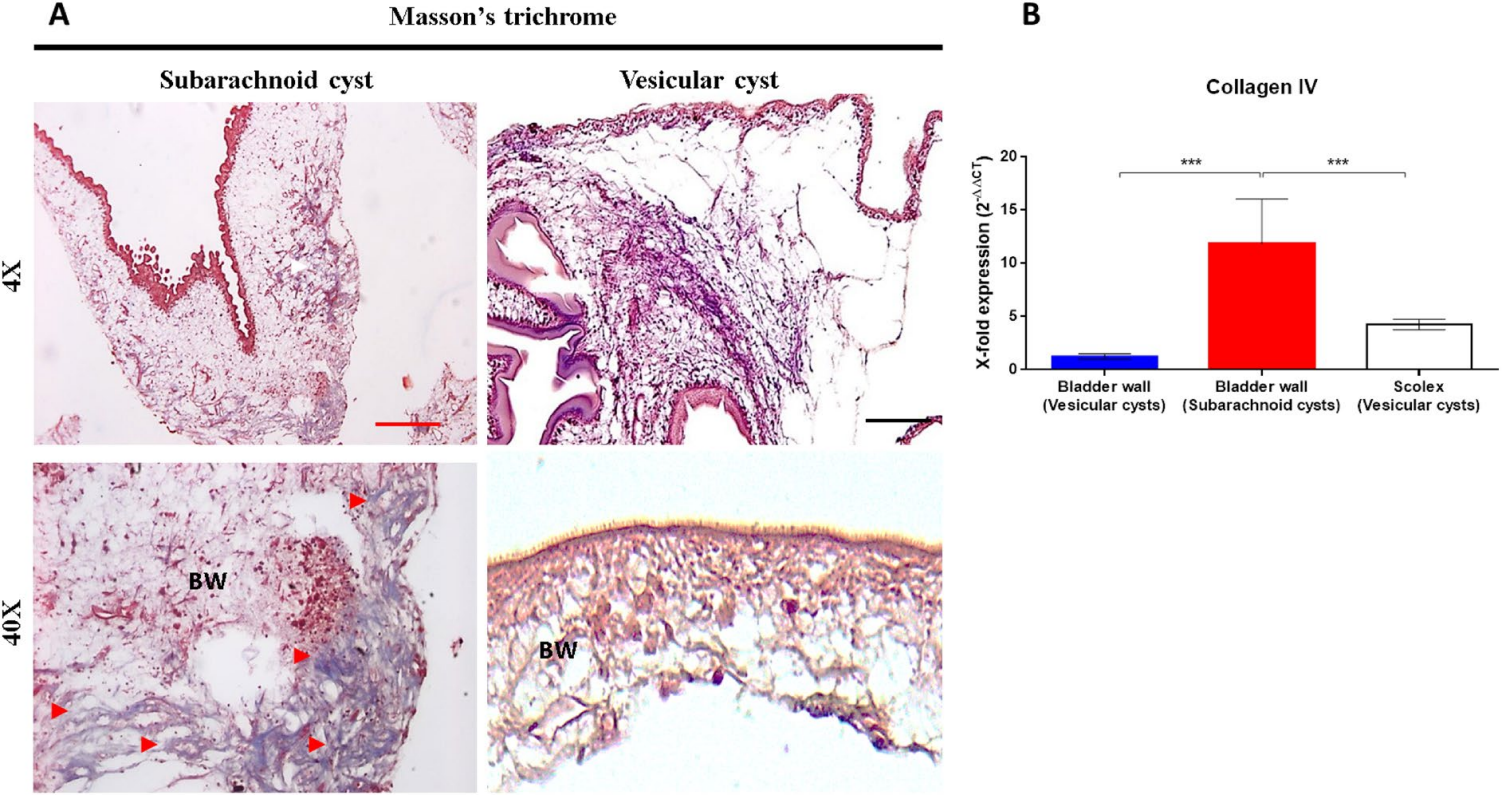
Increased expression and synthesis of extracellular matrix components in the bladder wall of subarachnoid cysts. (**A**) Localization of collagen in the bladder wall of subarachnoid and vesicular cysts by Masson's trichrome stain. Light microscopy images of representative samples at ×4 (upper) and ×40 (lower); blue-stain collagen fibers were observed in the bladder wall of subarachnoid cysts (red arrowheads). Red scale bar: 200 µm, black scale bar: 100 µm. BW: Bladder wall. (**B**) Quantitative gene expression of *collagen IV* in subarachnoid and vesicular cysts. Significant overexpression was observed in the subarachnoid cysts. Statistically significant differences are indicated by asterisks (Mann–Whitney U test). Asterisks represent level of significance: ***p < 0.001.

Histological studies demonstrate that the bladder wall of the subarachnoid cysts is a disordered structure that also differs in the quantity of their main tissue components^33^. We validated changes in two cellular component categories: Extracellular matrix (GO: 0031012) and Collagen type IV trimer (GO: 0005587). This evaluation focused on assessing the expression and deposition of collagen, a major structural constituent of basement membranes, which plays diverse and crucial biological roles^34,35^. Masson’s trichrome staining showed a high amount of blue-stained collagen fibers in the bladder wall of subarachnoid cysts. Additionally, using quantitative PCR, we found a significant increase in the expression of the homolog *collagen IV* gene (TsM_000925000, *p* value ***: 0.0004) in subarachnoid cysts compared to vesicular form (Fig. 3). These findings demonstrate the aberrant morphology and tissue component alterations in the subarachnoid cyst.

### The bladder wall of subarachnoid cysts shows increased lipid uptake and a higher metabolism

**Figure 4:**
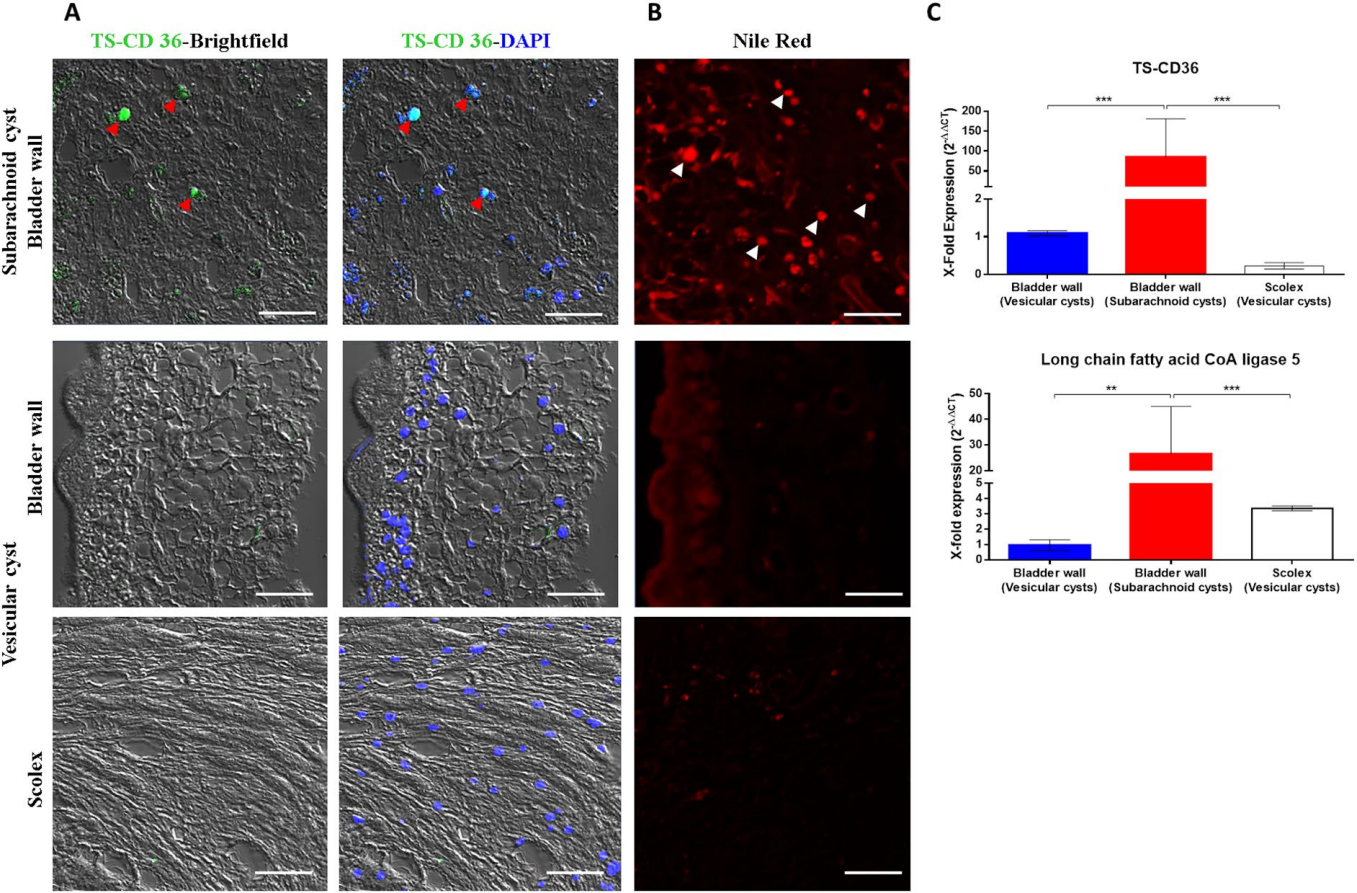
Increased lipid uptake and metabolism in the bladder wall of subarachnoid cysts. (**A**) Localization of TSCD36 in the bladder wall of the subarachnoid (upper) and vesicular cysts (middle and lower) by IF. Confocal microscopy images of representative samples; positive cells were observed only in the bladder wall of subarachnoid cysts (red arrowheads). White scale bar: 20 µm, ×20, zoom4. (**B**) Identification of lipid droplets in the bladder wall of the subarachnoid (upper) and vesicular cysts (middle and lower) by Nile red staining. Confocal microscopy images of representative samples; a high amount of lipid droplets were observed in the bladder wall of subarachnoid cysts (white arrowheads). White scale bar: 20 µm, ×20, zoom4. (**C**) Quantitative gene expression of *tscd36*, and *long chain fatty acid CoA ligase 5* in subarachnoid and vesicular cysts. Significant overexpression in the evaluated genes was observed in the subarachnoid cysts. Statistically significant differences are indicated by asterisks (Mann–Whitney U test). Asterisks represent level of significance: ***p < 0.001; **p < 0.01.

Parasitic helminths are incapable of de novo fatty acid biosynthesis^36,37^; they have developed specialized strategies to ensure the uptake and transport of lipids from the host tissue, like the release of fatty acid-binding proteins (FABP)^38,39^. Interestingly, GO analysis revealed enrichment in processes related to uptake and lipid metabolism in the bladder wall of subarachnoid cysts. Categories associated to biological processes, such as lipid transport (GO: 0006869), sterol transport (GO: 0015918), and cellular sphingolipid homeostasis (GO: 0090156) exhibited upregulation. In addition, flippase and floppase activities (GO: 0140327; GO: 0140328) belonging to the molecular function also displayed upregulation (Supplementary datas 3, 4). Subarachnoid cysts display significant lipid droplets in their bladder wall by Red Nile staining (Fig. 4B), indicating the presence of lipids which include triglycerides, cholesterol, and phospholipids.

Among the overexpressed genes relevant to lipid uptake, we identified the sequence of the homolog *cd36* gene, TS-CD36 (TsM_000162100), a scavenger receptor that allows the translocation of long-chain fatty acids^40^. Using IF and quantitative PCR analyses, we detected TS-CD36-positive cells distributed across the bladder wall of the subarachnoid cysts (Fig. 4A), accompanied by a significant overexpression of *ts-cd36* gene, (*p* value ***: 0.0004) (Fig. 4C). We also observed a significant increase in the expression levels of *long-chain fatty acid CoA ligase 5* (TsM_000751900, *p* value **: 0.0091; ***: 0.0004), which catalyzes the conversion and activation of long-chain fatty acids into fatty acyl-CoA, essential for syntheses of cellular lipids and degradation via beta-oxidation^41^.

On the other hand, subarachnoid cysts show enrichment in glucose import across the membrane (GO: 0098708), fucose metabolic process (GO: 0006004), and amino acid metabolic process (GO: 1901605). Additionally, water transport (GO: 0006833) is mainly promoted by the overexpression of aquaporin-4 (TsM_000078700, *p*-adj = 0.009).

### The subarachnoid cysts show an overexpression of detoxification systems

Cestodes, like other parasites, have developed diverse detoxification mechanisms to solubilize and excrete various compounds, including drugs^42^. We observed a significant increase in the expression levels of genes encoding enzymes with detoxification and antioxidant activities. This includes overexpression in glutathione-S-transferases µ-class (TsM_000639300, *p*-adj = 2.75 × 10^–6^; TsM_00137400, *p*- adj = 7.36 × 10^–9^), thioredoxins (TsM_000800400, *p*-adj = 0.0335; TsM_000819900, *p*-adj = 0.0309), glutaredoxin Fe-S (TsM_000502600, *p*-adj = 0.047), copper-zinc superoxide dismutase (TsM_000891800, *p*-adj = 8 × 10^–4^), and selenoproteins (TsM_000301800, *p*-adj = 0.031; TsM_000685100, *p*-adj = 0.0075) in the bladder wall of subarachnoid cysts, compared to vesicular form (Supplementary datas 1, 2).

## Discussion

Tapeworms exhibit a remarkable capacity for proliferation, crucial for their transformation from ingested cysts to fully developed tapeworms and the development of proglottids^43^. Within cestodes, cyst proliferation serves as a means to increase their numbers in a host^44^. For instance, *Taenia crassiceps*, a close relative of *T. solium*, employs asexual budding to reproduce identical cysts, while the cyst of *Echinococcus multilocularis* can abnormally proliferate in humans, occasionally metastasizing to other organs. The rupture of *E. granulosus* cysts can disseminate throughout the peritoneum in both humans and animals. Immunosuppressed individual may harbor abnormally proliferating cestode cysts from various species^45^.

The mechanisms driving cestode proliferation and disease in humans and animals are complex^42^. They include parasite growth, which may consist in normal or abnormal proliferation, tissue interactions, cell communication mediated by host-derived growth factors, and the suppression of host immune responses. However, the aberrant proliferation of subarachnoid cysts in humans lacks a purposeful, helpful biological role in the parasite’s life cycle. Despite its relatively common occurrence in human infections^4,6,10^, the processes governing the development, growth, and regulation of subarachnoid cysts remain largely unexplored, although germinative cells are likely involved in controlling transformation and growth in cestodes^22,30,31^.

*T. solium*, like other cestodes, establishes intricate communication with host cells^46,47^. This interaction occurs through the interplay of biomolecules, including peptides, hormones, and cytokines of the host, and receptors located on the parasite’s surface^48–51^. We identified signaling pathways pivotal for the proliferation of the bladder wall. These pathways are activated by insulin, EGF, TGF-β, neuropeptides, and platelet-derived growth factor that act together to promote the abnormal growth of the parasite (Fig. 1 and Supplementary data 3, 4). It is evident that the parasite’s tissue is exposed and responds to a diverse array of host biomolecules. Many of these activate pathways sharing common intracellular mediators, suggesting the crucial role of these conserved pathways in stimulating parasite proliferation (Fig. 2). The integration of host signals by the parasite appears to be a very complex phenomenon that involves active participation of the germinative cells.

Subarachnoid cysts face the challenge of developing and growing within an environment limited in nutrients and growth factors. The concentration of micro and macromolecules often exhibit variations between CSF and blood^52,53^. The levels of these molecules notably differ due to their distinct roles and mechanisms of action in different body compartments. In this context, elevated receptor expression in subarachnoid cysts constitutes an adaptive strategy. This increased expression optimizes the activity of these pathways by enhancing the uptake of host molecules, allowing the cysts to overcome the limitations of growth factors and nutrients in their microenvironment.

This premise finds support in studies conducted on *E. multilocularis* using the yeast two-hybrid system^54^. This study demonstrate minimal or no interaction between the parasite insulin receptors and their endogenous ligands. Conversely, the ligand-binding domains located on the parasite insulin receptors exhibit strong interactions with human pro-insulin and emphasize the role of host insulin as the major ligand during the parasite’s developmental phase within the intermediate host. On the other hand, in the genomic analysis of 30 platyhelminth species, the TNF receptor was detected, while homologous TNF ligands were detected only in five free-living species, suggesting that parasitic platyhelminths might respond to TNF-related signals upon binding the host ligand^55^. Future studies evaluating the effects of host TNF-α on *T. solium* cyst will provide valuable insight into understanding the interplay mediated by this cytokine and the benefits it generates in the parasite.

**Figure 5:**
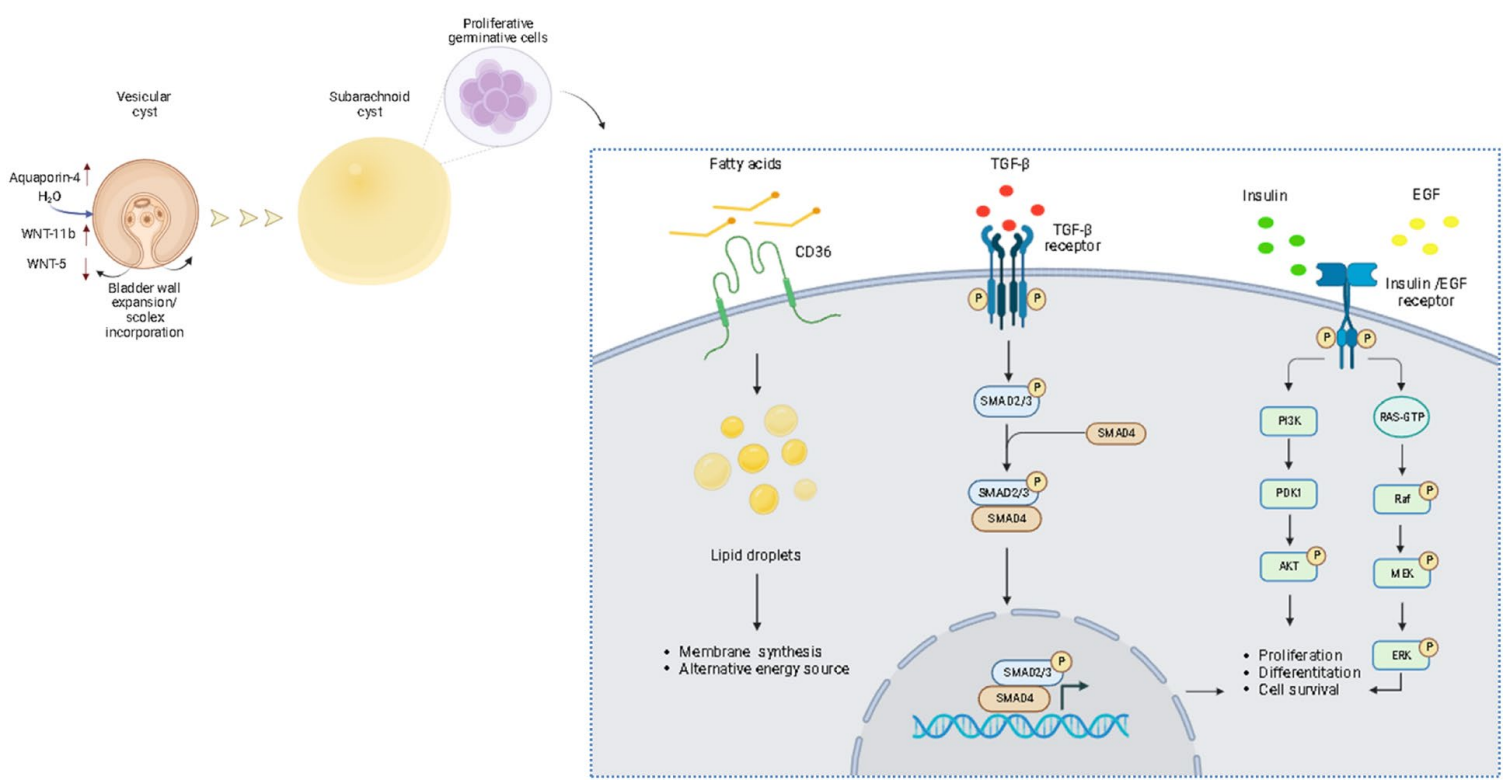
Schematic representation of molecular changes associated with cell proliferation through the activation of signaling pathways, and the uptake/metabolism of lipids in subarachnoid cysts of *T. solium*.

The WNT signaling pathway plays a critical role during the development and proliferation of platyhelminths^56^. Specific WNT ligands show localized expression on each A/P axis during larval development^26^. The loss of scolex in subarachnoid cysts constitutes the absence of the anterior axis that directs future adult-stage formation. However, the presence of germinative cells distributed throughout the bladder wall suggests an assimilation or incorporation of the germinative cells from the scolex into the bladder wall which allows the development and growth of subarachnoid cysts. The expansion of the bladder wall tissue, potentially facilitated by increased water influx due to the overexpression of aquaporins, WNT 11 and WNT 5 could be associated with this process (Fig. 5). Further studies are required to elucidate the specific events that trigger scolex assimilation.

Our findings reveal an increased activity associated with lipid uptake and transport, encompassing phospholipids and cholesterol in the subarachnoid cysts (Fig. 4 and Supplementary data 3, 4). Interestingly, lipid efflux within the parasite is not just into the cells; we observed an elevation in flippase and floppase activities, indicating increased lipid movement across the lipid bilayer. The notable overexpression of receptors or translocators like TSCD36 in the bladder wall of these cysts amplifies their capacity to efficiently capture and store lipids (Fig. 4). Consequently, the parasite fulfills its need for synthesizing new membranes crucial for the proliferation and growth of the bladder wall tissue.

In vitro studies using *T. crassiceps* cysticerci reveal that lipids serve as an alternative energy source for parasites when faced with low glucose levels or the presence of anthelmintics^57,58^. Benzimidasole-based compounds, acting by inhibiting microtubule polymerization or assembly, disrupt tubulin polymerization, consequently affecting the uptake and transportation of nutrients like glucose. This disruption promotes the use of fatty acids for energy production. The presence of lipid droplets in the subarachnoid cyst (Fig. 4B) probably represents an adaptation of the parasite to an environment relatively deficient in growth factors or nutrients. This adaptation, together with the overexpression of detoxification proteins, could account for the reduced effectiveness of conventional anthelmintic treatments in cases of SANCC.

In this context, we identify potential therapeutic targets including TS-CD36 and the EGF/TGF-β signaling pathways associated with cell proliferation (Fig. 5). These findings broaden the scope for exploring novel drugs or treatments aimed at regulating parasite proliferation. TS-CD36 emerges as an attractive target given its multifaceted involvement in cell biology^40^.

SANCC is associated with an increased regulatory immune response, resulting in an anti-inflammatory profile characterized by high levels of IL10^59^. However, further studies are required to perform a thorough protein characterization of subarachnoid cysts to identify the key protein mediators secreted by the parasite and the signaling pathways activated in the host tissue.

Our findings align with prior research conducted in other parasite species^16,17,60–63^. The ORF strain of *T. crassiceps* cysts typically reproduces through asexual budding. This proliferative characteristic has allowed the established of an experimental model of SANCC^64^. Our findings are in line with the transcriptomic profile of *T. crassiceps*, which show an increase in extracellular matrix components^65^. A comparative study at a molecular level between *T. solium* subarachnoid cysts and *T. crassiceps* cysts would provide valuable information about the main pathways associated with proliferation that are conserved in both species. It is important to note that the annotation of all *T. solium* genes remains incomplete. Additionally, the protein products of several differentially expressed genes remain unidentified, hindering the assignment of specific biological function. Despite these limitations, our analyses have successfully revealed genes and signaling pathways that promote parasite proliferation. These insights shed light on adaptations to the microenvironment that the parasite undergoes.

## Materials and methods

### Experimental design

A total of 12 subarachnoid cysts and 11 vesicular cysts samples of *T. solium* were used to identify alterations at the RNA expression levels. Total RNA was analyzed by RNAseq. Changes in the expression of some transcripts were validated by quantitative PCR, histological staining, and immunofluorescence. Additionally, primary cell cultures of germinative cells were established to evaluate in vitro the action of transforming growth factor beta (TGF-β) and epidermal growth factor (EGF). We confirm that the current study is reported in accordance with ARRIVE guidelines^66^.

### Ethics statement

The approval for the use of racemose cyst samples was obtained from de Ethics Committee of Instituto Nacional de Ciencias Neurológicas (INCN) (242-2018-DG-INCN). Additionally, the protocols for the use of animals were reviewed and approved by the Institutional Ethics Committees for the Use of Animals of the Veterinary School of Universidad Nacional Mayor de San Marcos (Protocol number 006) in Lima, Peru. All methods were carried out in accordance with relevant guidelines and regulations.

### Sample collection

Sections of subarachnoid cysts were obtained post-surgical extraction from human patients at the INCN surgical ward in Lima, Peru. These samples were anonymized and then transported to the laboratory in phosphate buffer solution (PBS) pH 7.4 (Gibco-Invitrogen, Gaithersburg, MD, USA). Samples of vesicular cyts were obtained from three naturally infected pigs from an endemic zone of Peru^67^. The pigs were anesthetized by intramuscular injection of ketamine (11 mg/kg) and xylazine (2 mg/kg). The animals were euthanized with a lethal dose of pentobarbital (9 mg/kg), the parasites were removed from the skeletal muscle and transported to the laboratory in PBS pH 7.4 supplemented with antibiotics (100 U/ml penicillin and 100 g/ml streptomycin from Gibco-Invitrogen, Gaithersburg, MD, USA). The samples were fixed in neutral buffered formalin and paraffin-embedded for histology, preserved in RNAlater (Qiagen, Hiden, Germany) at − 70 °C, and or used for cell isolation.

### Total RNA isolation and sequencing

A total of 6 cyst samples (three subarachnoid and three vesicular cyst samples) were used for total RNA isolation. The vesicular cyst samples were separated into the bladder wall and scolex with a scalpel, and nucleic acids were isolated from both structures separately. Total RNA from each sample was extracted using Qiagen RNeasy Plus Universal mini kit (Qiagen, Hiden, Germany) and treated with TURBO DNase (Thermo Fisher Scientific, Waltham, MA, USA). RNA samples were quantified using Qubit 2.0 Fluorometer (Life Technologies, Carlsbad, CA, USA) and the integrity was checked using Agilent TapeStation 4200 (Agilent Technologies, Palo Alto, CA, USA).

The samples were prepared for rRNA depletion using the QIAseq FastSelect rRNA HMR Kit (Qiagen, Hilden, Germany). RNA sequencing library preparation employs the NEBNext Ultra II RNA Library Preparation Kit for Illumina and the manufacturer’s recommendations were followed (NEB, Ipswich, MA, USA). Briefly, enriched RNAs were fragmented for 15 min. at 94 °C. First strand and second strand cDNA were subsequently synthesized. cDNA fragments were then end repaired and the 3ʹ ends adenylated, and universal adapters ligated to cDNA fragments, followed by index addition and library enrichment with limited cycle PCR. Sequencing libraries were validated using the Agilent Tapestation 4200 (Agilent Technologies, Palo Alto, CA, USA), and quantified using Qubit 2.0 Fluorometer (ThermoFisher Scientific, Waltham, MA, USA) as well as by quantitative PCR (KAPA Biosystems, Wilmington, MA, USA). The sequencing libraries were multiplexed and clustered on a flowcell, then loaded on the Illumina instrument (HiSeq 4000) according to manufacturer’s instructions. The samples were sequencing using a 2 × 150 bp Pared End configuration. Image analysis and base calling were conducted by the Control software. Raw sequence data generated were converted into fastq files and de-multiplexed using Illumina’s bcl2fastq 2.17 software. One mismatch was allowed for index sequence identification.

### Data analysis

The RNA-Seq reads were subject to quality control with FastQC v0.11.5. Then quality filtering and adapter trimming were performed with BBDUK2 from BBMAP package v37.02, using the following settings: qtrim = w, trimq = 20, maq = 10, k = 23, mink = 11, hdist = 1, tbo, tpe, minlength = 100, removeifeitherbad = t. After that, genes and transcripts expression values were calculated using RSEM^68^ v1.2.30 and Bowtie 2^69^ as reads aligner. The reads were mapped against *T. solium* transcriptome obtained from WormBase.

(taenia_solium.PRJNA170813.WBPS16.mRNA_transcripts.fa.gz). Differential expression analysis was done using DESeq2^70^ v1.30.1. Two comparisons were performed: bladder wall of the subarachnoid cyst (group R) vs. bladder wall of the vesicular cyst (group V), as well as group R vs. scolex of the vesicular cyst (group N). It was required that differentially expressed genes have the adjusted *p*-value < 0.05. This was followed by gene set enrichment analysis (GSEA) with goseq from Bioconductor/R. Only hits with *p*-value < 0.05 were kept. Enrichment in Gene Ontology (GO) terms and Kyoto Encyclopedia of Genes and Genomes (KEGG) pathways were done separately. The KEGG and GO assignments to *T. solium* genes came from own annotation obtained with Trinity v2.8.4. The data generated during and/or analyzed during the current study are available in the GEO repository under accession number GSE266860.

### Validation by quantitative PCR

Real-time PCR was performed using 7 tissue samples (three subarachnoid and four vesicular cyst samples). The vesicular cysts were divided into the bladder wall and scolex as previously described^22^. All the samples were homogenized in 1 ml of TRIzol reagent (Invitrogen, Carlsbad, CA, USA) for total RNA isolation, the final concentrations were determined using a UV spectrophotometer (Nanodrop Products, Wilmington, DE, USA). cDNA was generated from 500 ng of total RNA using the High-Capacity cDNA Reverse Transcription Kit with MultiScribe RT polymerase and random primers (Applied Biosystems, Foster City, CA, USA) in a final volume of 20 µl per reaction with a protocol cycle of 10 min at 25 °C followed by 60 min at 37 °C, 5 min at 95 °C on SimpliAmp Thermal Cycler (Applied Biosystems, Foster City, CA, USA). Quantitative PCR (qPCR) was performed in 10 µl reaction volumes using SsoAdvanced Universal SYBR Green Supermix (BioRad Laboratories, Hercules, CA, USA) with designed primers (Supplementary data 5). qPCR reactions, run in triplicate, used the following cycling parameters: pre-incubation of 2 min at 50 °C and 10 min at 95 °C followed by 40 cycles of 15 s at 95 °C and 1 min at 60 °C, on a Lightcycler 96 System (Roche, Basel, Switzerland). The bladder wall of vesicular cyst was used as a calibration sample and we expressed the results as relative to the expression of the *gapdh* gene using the 2^−ΔΔCT^ formula^71^.

### Immunofluorescence (IF)

The formalin-fixed paraffin-embedded (FFPE) samples of three subarachnoid and four vesicular cysts were cut into 4 µm sections placed on poly-l-lysine coated slides and treated as previously described^22,23^. The slides were submerged in 10 mM citrate buffer (10 mM citric acid, 0.05% Tween 20, pH 6.0) for 30 min at 95 °C, incubated for 30 min with a blocking solution (PBS pH 7.2, 0.05% Tween 20, 0.1% Triton X-100, 2% goat serum, 2% BSA) in a humid chamber at RT, then incubated overnight at 4 °C with primary antibody in PBS (Supplementary data 5), washed three times for 2 min with washing solution (PBS pH 7.2, 0.05% Tween 20) and then incubated for 30 min at RT with the fluorescein-labeling goat anti-rabbit antibody (Jackson ImmunoResearch Lab, West Grove, PA, USA) diluted 1/500 in PBS. The sections were then washed with PBS and mounted with VectaShield mounting medium with DAPI (Vector, Laboratories, Burlingame, CA, USA). Images were captured by confocal microscopy (Zeiss, LSM880, Oberkochen, Germany).

### Masson’s trichrome and Nile Red staining

Sections of FFPE samples were deparaffinized and stained using Masson's Trichrome staining kit (ThermoFisher Scientific, Waltham, MA, USA) to identify collagen fibers, and Nile Red staining kit (Abcam, Cambridge, UK) for the fluorometric detection of intracellular lipid droplets using fluorescence microscopy (excitation/emission: 550/640 nm). All the protocols were performed according to manufacturer’s instructions.

### Cell isolation and in vitro evaluations

Portions of three subarachnoid cysts were used for cellular isolation. The cell cultures were established as previously described^22^. The cells were resuspended, and an aliquot of each cell suspension was diluted for cell counting and viability evaluation by Trypan blue dye exclusion. We seeded 20,000 cells in 24-well plates; the action of TGF beta at 0.01 ng/ mL^30^ and EGF at 100 ng/ mL^31^ were evaluated in triplicate separately or in combination, and the cell count was performed every 24 h^22^.

### Fluorescence in situ hybridization (FISH)

Prior published protocols were employed with some modifications^22^. The cells isolated from subarachnoid cysts were fixed with chilled methanol overnight at 4 °C and incubated at 56 °C with a digoxigenin-labeled LNA type probe (*argo2*; 5ʹTACGACAACGATGAGTTGGAGA-3ʹ) (Qiagen/Life Technologies, Gaithersburg, MD) at a concentration of 0.5 ng/μl designed using the annotated sequence of the gene (TsM_000674100). The samples were incubated overnight at 4 °C with an anti-digoxigenin antibody (1/500) conjugated to peroxidase (Roche) and placed 10 min. in a fluorescein-tyramide developing solution. For phospho-SMAD2 protein detection, cells were incubated overnight at 4 °C with an anti-phospho SMAD2 antibody (1/500). Then for 30 min at RT with the fluorescein-labeling goat anti-rabbit antibody (Jackson ImmunoResearch Lab, West Grove, PA, USA) diluted 1/500 in PBS. Finally, the cells were mounted with VectaShield mounting medium with DAPI (Vector, Laboratories, Burlingame, Ca). Images were captured by confocal microscopy (Zeiss, LSM880, Oberkochen, Germany).

### Statistical analysis

Statistical analysis employed Mann–Whitney U test for two groups, were calculated using Prism software (Graphpad, San Diego, CA) for comparisons of the gene expression between bladder wall of subarachnoid cysts vs. bladder wall of vesicular cysts or bladder wall of subarachnoid cysts vs. scolex of vesicular cysts. Differences with *p* values < 0.05 were considered statistically significant. The growth curves are presented as a dot plot with the mean ± standard deviation. Quantitative comparisons of EGF and TGF-β effects in cell proliferation were analyzed by one-way ANOVA and *p* values < 0.05 were considered statistically.

### Supplementary Information

Supplementary Information 1.

Supplementary Information 2.

Supplementary Information 3.

Supplementary Information 4.

Supplementary Information 5.

Supplementary Figure 1.

Supplementary Figure 2.

## Data Availability

The data generated during and/or analyzed during the current study are available in the GEO repository under accession number GSE266860.
