# Supplementary material for: Transcriptomic analysis of subarachnoid cysts of Taenia solium reveals mechanisms for uncontrolled proliferation and adaptations to the microenvironment
Source: Sci Rep. 2024 May 23;14:11833. doi: 10.1038/s41598-024-61973-9 (PMC11116493; doi:10.1038/s41598-024-61973-9)
Supplement: Supplementary file 6 — Supplementary Figure 1. [file 41598_2024_61973_MOESM6_ESM.pdf]

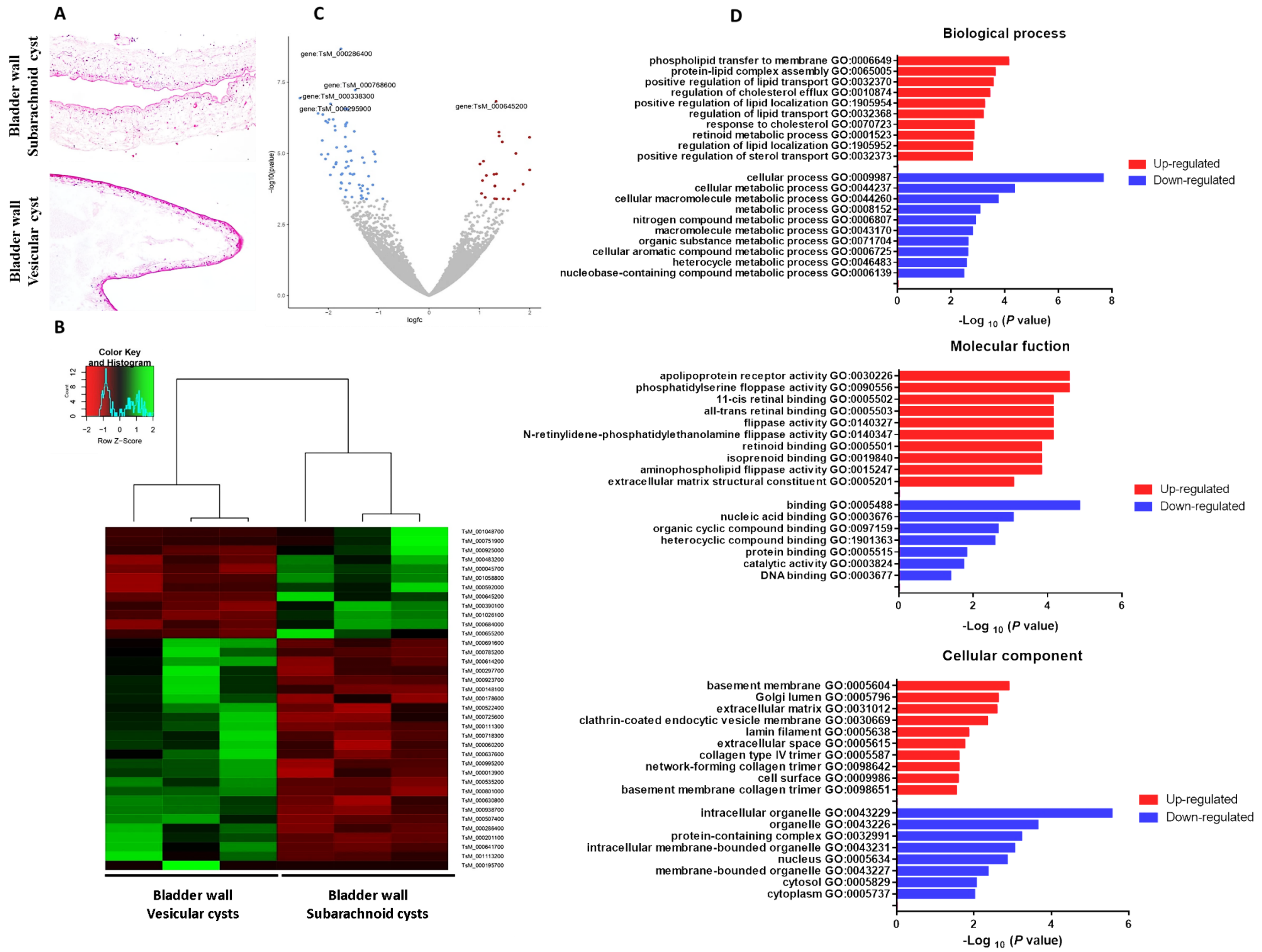

A
Bladder wall Subarachnoid cyst
Bladder wall Vesicular cyst
C
gene:TsM_000286400
gene:TsM_000768600
gene:TsM_000338300
gene:TsM_000295900
gene:TsM_000645200
-log10(pvalue)
logfc
B
Color Key and Histogram
Count
Row Z-Score
Bladder wall Vesicular cysts
Bladder wall Subarachnoid cysts
D
Biological process
phospholipid transfer to membrane GO:0006649
protein-lipid complex assembly GO:0065005
positive regulation of lipid transport GO:0032370
regulation of cholesterol efflux GO:0010874
positive regulation of lipid localization GO:1905954
regulation of lipid transport GO:0032368
response to cholesterol GO:0070723
retinoid metabolic process GO:0001523
regulation of lipid localization GO:1905952
positive regulation of sterol transport GO:0032373
cellular process GO:0009987
cellular metabolic process GO:0044237
cellular macromolecule metabolic process GO:0044260
metabolic process GO:0008152
nitrogen compound metabolic process GO:0006807
macromolecule metabolic process GO:0043170
organic substance metabolic process GO:0071704
cellular aromatic compound metabolic process GO:0006725
heterocycle metabolic process GO:0046483
nucleobase-containing compound metabolic process GO:0006139
Up-regulated
Down-regulated
-Log 10 (P value)
Molecular fuction
apolipoprotein receptor activity GO:0030226
phosphatidylserine floppase activity GO:0090556
11-cis retinal binding GO:0005502
all-trans retinal binding GO:0005503
flippase activity GO:0140327
N-retinylidene-phosphatidylethanolamine flippase activity GO:0140347
retinoid binding GO:0005501
isoprenoid binding GO:0019840
aminophospholipid flippase activity GO:0015247
extracellular matrix structural constituent GO:0005201
binding GO:0005488
nucleic acid binding GO:0003676
organic cyclic compound binding GO:0097159
heterocyclic compound binding GO:1901363
protein binding GO:0005515
catalytic activity GO:0003824
DNA binding GO:0003677
Up-regulated
Down-regulated
-Log 10 (P value)
Cellular component
basement membrane GO:0005604
Golgi lumen GO:0005796
extracellular matrix GO:0031012
clathrin-coated endocytic vesicle membrane GO:0030669
lamin filament GO:0005638
extracellular space GO:0005615
collagen type IV trimer GO:0005587
network-forming collagen trimer GO:0098642
cell surface GO:0009986
basement membrane collagen trimer GO:0098651
intracellular organelle GO:0043229
organelle GO:0043226
protein-containing complex GO:0032991
intracellular membrane-bounded organelle GO:0043231
nucleus GO:0005634
membrane-bounded organelle GO:0043227
cytosol GO:0005829
cytoplasm GO:0005737
Up-regulated
Down-regulated
-Log 10 (P value)

**Supplementary Fig1. Identification of differentially expressed genes in the bladder wall of subarachnoid cysts compared to vesicular cysts. (A)** Representative images of tissue samples used in the first comparison (bladder wall of the subarachnoid and vesicular cysts). **(B)** Hierarchical clustering of RNAseq samples based on gene expression patterns in the bladder wall of the subarachnoid and vesicular cysts. The color scale for the figure, as indicated by the scale bar in the upper left corner of Fig. 1b, indicates log2 fold changes from −2 to +2. Data used to generate the heatmap is included in Supplementary Data 1. **(C)** Volcano plot of gene-expression pattern vs. the adjusted *p*-value, with the five most differentially expressed genes indicated. **(D)** Gene ontology enrichment analysis for biological process, molecular function, and cellular component. Upregulated categories are in red bars and downregulated in blue. Data used to generate the graphics is included in Supplementary Data 3.
