# Supplementary material for: Transcriptomic analysis of subarachnoid cysts of Taenia solium reveals mechanisms for uncontrolled proliferation and adaptations to the microenvironment
Source: Sci Rep. 2024 May 23;14:11833. doi: 10.1038/s41598-024-61973-9 (PMC11116493; doi:10.1038/s41598-024-61973-9)
Supplement: Supplementary file 7 — Supplementary Figure 2. [file 41598_2024_61973_MOESM7_ESM.pdf]

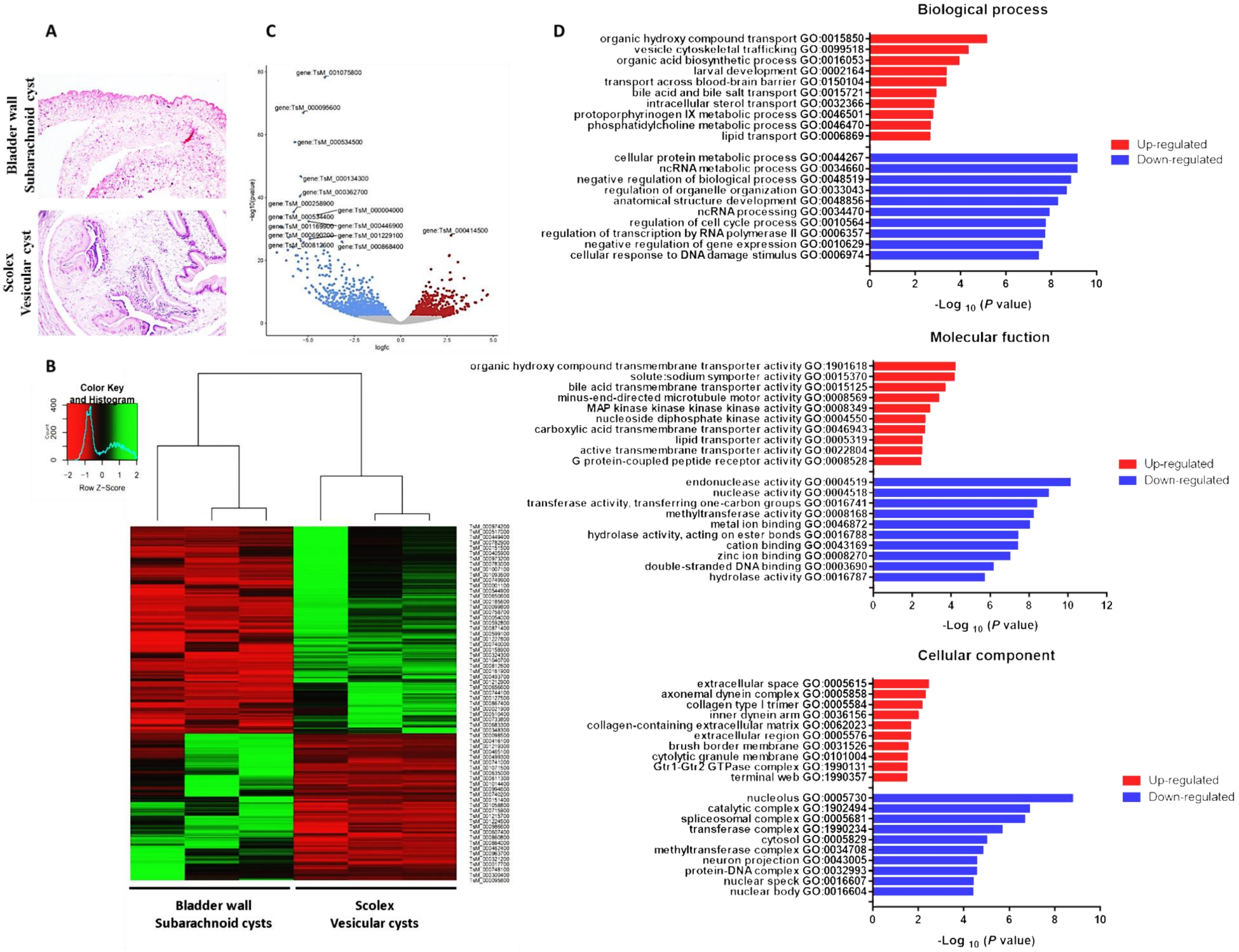
A
Bladder wall
Subarachnoid cyst
Scolex
Vesicular cyst
C
gene:TsM_001075800
gene:TsM_000095600
gene:TsM_000534500
gene:TsM_000134300
gene:TsM_000362700
gene:TsM_000258900
gene:TsM_000004000
gene:TsM_000534400
gene:TsM_000446900
gene:TsM_001229100
gene:TsM_000868400
gene:TsM_000414500
-log10(pvalue)
logfc
B
Color Key
and Histogram
Count
Row Z-Score
Bladder wall
Subarachnoid cysts
Scolex
Vesicular cysts
D
Biological process
organic hydroxy compound transport GO:0015850
vesicle cytoskeletal trafficking GO:0099518
organic acid biosynthetic process GO:0016053
larval development GO:0002164
transport across blood-brain barrier GO:0150104
bile acid and bile salt transport GO:0015721
intracellular sterol transport GO:0032366
protoporphyrinogen IX metabolic process GO:0046501
phosphatidylcholine metabolic process GO:0046470
lipid transport GO:0006869
cellular protein metabolic process GO:0044267
ncRNA metabolic process GO:0034660
negative regulation of biological process GO:0048519
regulation of organelle organization GO:0033043
anatomical structure development GO:0048856
ncRNA processing GO:0034470
regulation of cell cycle process GO:0010564
regulation of transcription by RNA polymerase II GO:0006357
negative regulation of gene expression GO:0010629
cellular response to DNA damage stimulus GO:0006974
Up-regulated
Down-regulated
-Log 10 (P value)
Molecular fuction
organic hydroxy compound transmembrane transporter activity GO:1901618
solute:sodium symporter activity GO:0015370
bile acid transmembrane transporter activity GO:0015125
minus-end-directed microtubule motor activity GO:0008569
MAP kinase kinase kinase kinase activity GO:0008349
nucleoside diphosphate kinase activity GO:0004550
carboxylic acid transmembrane transporter activity GO:0046943
lipid transporter activity GO:0005319
active transmembrane transporter activity GO:0022804
G protein-coupled peptide receptor activity GO:0008528
endonuclease activity GO:0004519
nuclease activity GO:0004518
transferase activity, transferring one-carbon groups GO:0016741
methyltransferase activity GO:0008168
metal ion binding GO:0046872
hydrolase activity, acting on ester bonds GO:0016788
cation binding GO:0043169
zinc ion binding GO:0008270
double-stranded DNA binding GO:0003690
hydrolase activity GO:0016787
Up-regulated
Down-regulated
-Log 10 (P value)
Cellular component
extracellular space GO:0005615
axonemal dynein complex GO:0005858
collagen type I trimer GO:0005584
inner dynein arm GO:0036156
collagen-containing extracellular matrix GO:0062023
extracellular region GO:0005576
brush border membrane GO:0031526
cytolytic granule membrane GO:0101004
Gtr1-Gtr2 GTPase complex GO:1990131
terminal web GO:1990357
nucleolus GO:0005730
catalytic complex GO:1902494
spliceosomal complex GO:0005681
transferase complex GO:1990234
cytosol GO:0005829
methyltransferase complex GO:0034708
neuron projection GO:0043005
protein-DNA complex GO:0032993
nuclear speck GO:0016607
nuclear body GO:0016604
Up-regulated
Down-regulated
-Log 10 (P value)

Supplementary Fig. 2. Identification of differentially expressed genes in the bladder wall of subarachnoid cysts compared to the scolex of vesicular cysts. (A) Representative images of tissue samples used in the second comparison (bladder wall of the subarachnoid cysts and the scolex of vesicular cysts). **(B)** Hierarchical clustering of RNAseq samples based on gene expression patterns in the bladder wall of the subarachnoid and scolex of vesicular cysts. The color scale for the figure, as indicated by the scale bar in the upper left corner of Fig. 2b, indicates log2 fold changes from −2 to +2. Data used to generate the heatmap is included in Supplementary Data 2. **(C)** Volcano plot of gene-expression pattern vs. the adjusted *p*-value, with the fifteen most differentially expressed genes indicated. **(D)** Gene ontology enrichment analysis for biological process, molecular function, and cellular component. Upregulated categories are in red bars and downregulated in blue. Data used to generate the graphics is included in Supplementary Data 4.
